# Extracellular Matrix Membrane Induces Cementoblastic/Osteogenic Properties of Human Periodontal Ligament Stem Cells

**DOI:** 10.3389/fphys.2018.00942

**Published:** 2018-07-18

**Authors:** Yuanyuan Wang, Silvana Papagerakis, Denver Faulk, Stephen F. Badylak, Yuming Zhao, Lihong Ge, Man Qin, Petros Papagerakis

**Affiliations:** ^1^Department of Pediatric Dentistry, School and Hospital of Stomatology, Peking University, Beijing, China; ^2^Department of Surgery, College of Medicine, University of Saskatchewan, Saskatoon, SK, Canada; ^3^Department of Otolaryngology, Medical School, University of Michigan, Ann Arbor, MI, United States; ^4^McGowan Institute for Regenerative Medicine, University of Pittsburgh, Pittsburgh, PA, United States; ^5^Department of Surgery, University of Pittsburgh, Pittsburgh, PA, United States; ^6^Department of Bioengineering, University of Pittsburgh, Pittsburgh, PA, United States; ^7^Colleges of Dentistry and Biomedical Engineering, Toxicology, Pharmacy, Nutrition, Anatomy and Cell Biology, University of Saskatchewan, Saskatoon, SK, Canada; ^8^Department of Orthodontics and Pediatric Dentistry, School of Dentistry, University of Michigan, Ann Arbor, MI, United States

**Keywords:** periodontal ligament stem cells, periodontal diseases, extracellular matrix, scaffolds, tissue regeneration

## Abstract

**Objective:** Periodontitis affects nearly 90% of adults over the age of 70, resulting to periodontal tissue infection, destruction, and ultimately tooth loss. Guided tissue regeneration (GTR) is a method widely used to treat severe periodontal disease, and involves placement of an occlusive barrier to facilitate regeneration of the damaged area by periodontal ligament stem cells (PDLSCs). In this study, we evaluate natural extracellular matrix (ECM) as a scaffold material to provide a suitable microenvironment to support the proliferation, differentiation, and tissue-regenerating properties of PDLSCs.

**Design:** The viability, proliferation, apoptosis, and migration of PDLSCs cultured on ECM membrane, that was isolated from porcine urinary bladders, were compared with those cultured on type I collagen membrane, a commonly used scaffold in GTR. To evaluate the effects of ECM vs. type I collagen on the tissue-regenerating properties of PDLSCs, the bio-attachment and cementoblastic/osteogenic differentiation of PDLSCs were evaluated.

**Results:** Incubation of PDLSCs with ECM resulted in increased viability, proliferation, and reduced apoptosis, compared with type I collagen treated PDLSCs. Co-culture with ECM membrane also increased the migration and bio-attachment of PDLSCs. Incubation of PDLSCs with ECM membrane increased expression of the cementoblastic/osteogenic differentiation markers BSP, RUNX2, ALP, OPN, OCN, and periostin.

**Conclusion:** ECM membrane enhances the proliferation and regenerative properties of PDLSCs, indicating that ECM membrane can serve as a suitable scaffold in the application of GTR to treat periodontal disease.

## Introduction

Periodontitis is a major dental public health issue that results to periodontal tissues destruction and tooth loss (Haffajee and Socransky, 1994; Slots et al., 1999; Pihlstrom et al., 2005) affecting the majority of older adult population in the United States (Nakashima and Reddi, 2003; Pihlstrom et al., 2005; Nanci and Bosshardt, 2006).

Guided tissue regeneration (GTR) (Gottlow et al., 1986, 1990) is a widely used regenerative procedure for severe periodontal tissue destruction. GTR success relies on stem cell migration from the periodontal ligament (PDLSCs) into the damaged area and subsequent periodontal tissue regeneration.

Collagen Type I membranes (COLI) are often used as a barrier in GTR therapy, and have shown to facilitate PDLSCs migration, proliferation, and differentiation while inducing minimal cytotoxicity (Gentile et al., 2011) and enhancing hemostasis and wound healing (Postlethwaite et al., 1978). COLI membranes have been extensively used in clinical trials (Hämmerle et al., 1992; Mundell et al., 1993; Alarrayed et al., 1995; Bornstein et al., 2007; Zubery et al., 2007; Tal et al., 2008; Friedmann et al., 2011; Ivanovski et al., 2015). However, the fast COLI membrane degradation rate observed in many studies can produce loss of cell attachment (Rothamel et al., 2004) resulting in difficulties to retain structural integrity necessary for tissue regeneration. Therefore, appropriate alternative membranes that can enhance and sustain periodontal tissue regeneration under inflammatory conditions remains to be discovered.

To address this challenge, natural extracellular matrix (ECM) scaffolds are considered excellent alternatives to COLI membranes because of their regenerative and anti-inflammatory properties (Currie et al., 2001; Patino et al., 2002). The binding of ECM proteins to cell surface integrins and other receptors promotes cell survival, proliferation, and migration (Nelson and Bissell, 2006; Geiger and Yamada, 2011) resulting in successful tissue regeneration in clinical trials. ECM proteins have also been shown to successfully guide the differentiation of stem cells both *in vitro* (Li et al., 2004; Brennan et al., 2008) and *in vivo* (Beattie et al., 2009; Agrawal et al., 2011) while enhancing tissue regeneration in skeletal muscle (Marelli et al., 2010; Mase et al., 2010), esophagus (Nieponice et al., 2009), and urinary bladder (Boruch et al., 2010). ECM scaffolds are extracted and prepared from dermis, small intestine, urinary bladder, and pericardium (Gilbert et al., 2006). The shape and mechanical strength of ECM scaffolds can be customized by different processing techniques (Jadhav et al., 2006). We have recently showed that ECM scaffolds from bladder promote dental pulp mesenchymal stem cells differentiation and dental tissue regeneration (Wang et al., 2015).

Here, we comparatively evaluated the effects of natural ECM membranes and COLI membranes on the PDLSCs proliferation rate, osteogenic/cementoblastic differentiation, migration, and attachment. Our data suggests that ECM membranes could provide niche-like signals to PDLSCs and also that ECM membranes are superior in stimulating and accelerating the growth, attachment, and differentiation of human PDLSCs when compared to type I collagen membranes.

## Materials and Methods

### Cell Culture

Normal human impacted third molars (*n* = 5) were collected from adult patients (20–25-year-old man) at the oral surgery clinic of the University of Michigan School of Dentistry. All extracted teeth were used for isolating human PDLSCs. Totally five different teeth were collected and five different PDLSC primary cell lines were generated and examined following the exact same procedures and methods. Results obtained from the five different PDLSCs cultures were similar.

This study was performed under an ethics protocol previously approved by the Institutional Review Board Ethics Committee of the University of Michigan. All patients that donated their extracted teeth were provided written informed consent and all samples were collected without any identification.

Periodontal ligament stem cells were isolated according to a previously described method (Seo et al., 2004). Briefly, periodontal ligament tissue was gently separated from the surface of the tooth root, and subsequently digested in a solution of 3 mg/mL collagenase type I (Sigma, St. Louis, MO, United States) and 4 mg/mL dispase (Sigma, St. Louis, MO, United States) for 1 h at 37°C. Single-cell suspensions were obtained by passing the cells through a 70 μm strainer (Falcon). Single-cell suspensions (0.5–1.0 × 10^3^ cells/well) were seeded onto six-well plates (Costar) containing alpha modification of Eagle’s medium (REF:12571, GIBCO/BRL, Grand Island, NY, United States) supplemented with 15% fetal bovine serum (FBS; Hyclone), 100 U/mL penicillin and 100 mg/mL streptomycin (Sigma, St. Louis, MO, United States). Cultures were incubated at 37°C in 5% CO_2_. Human dental pulp stem cells (DPSCs) were kindly provided by the laboratory of Dr. Kaigler at the University of Michigan. We used DPSCs as a negative control for scleraxis expression. Scleraxis is a specific maker of tendon tissue and PDL and it was only expressed in PDLSCs but not in DPSCs. The PDLSCs used in this study were cells either from passage 2 or passage 3 after the primary culture initiation.

### ECM and Collagen Membrane Materials

Extracellular matrix membrane used in this study was provided by professor Badylak, and isolated from porcine urinary bladders as it has been descripted (Freytes et al., 2008). Type I collagen membranes were obtained from Zimmer Dental (Zimmer Dental, Carlsbad, CA, United States). First, cells were plated and attached on the bottom of the plates. Then, equal size of ECM or collagen I membranes were placed into each well and slowly emerged toward to bottom of each well.

### Evaluation of Human PDLSC Marker Expression

#### RT-PCR

Total RNA was isolated from PDLSCs and DPSCs using TRIzol reagent (Invitrogen, Carlsbad, CA, United States), and 2 μg of total RNA was reverse transcribed into cDNA with TaqMan reverse transcription reagents (Applied Biosystems, Branchburg, NJ, United States), following the manufacturer’s recommendations. The resulting cDNA was amplified by RT-PCR, using AmpliTaq Gold DNA Polymerase (Applied Biosystems). RT-PCR amplifications were performed the following thermal conditions: 95°C for 30 s, 60°C for 30 s, and 72°C for 30 s. The cycle number is 40. The reactions were performed using an ABI PRISM 7500 Sequence Detection System (Applied Biosystems, Foster City, CA, United States), using specific primers. Primers specific for scleraxis gene were used to evaluate the specificity of PDLSCs cultures since scleraxis is considered as PDLSCs lineage marker. Specific primers for bone sialoprotein (*IBSP*), runt-related transcription factor 2 (*RUNX2*), alkaline phosphatase (*ALPL*), osteopontin (*SPP1*), osteocalcin (*BGLAP*), and periostin (*POSTN*) were used for evaluating the cementoblastic/osteogenic differentiation of PDLSCs in our study. Relative RNA expression levels were normalized against Glyceraldehyde 3-phosphate dehydrogenase (GAPDH) reference gene. The design of the primers was based on published human cDNA sequences (**Table 1**). RT-PCR products were sub-cloned into pGEM-T Easy vector (Promega, Madison, WI, United States) and PCR product identity was confirmed by sequencing. Melting curve analysis was performed for all PCR reactions. Each experiment was repeated five times for each separate PDLSCs clone.

**Table 1:** Semi-quantitative Real-Time PCR primers.

Gene name	5′-sequence-3′	Product size (bp)	GenBank number
GAPDH	**Forward:** GCAAATTCCATGGCACCGTC	819	XM_003273723.2
	**Reverse:** GGTCCACCACCCTGTTGCTA		
scleraxis	**Forward:** CTCCAGCTACATCTCGCACC	220	NM_001008271.1
	**Reverse:** GCGGTCCTTGCTCAACTTTC		
ALP	**Forward:** GCGCAGGACAGGATTAAAGC	246	NM_014476.5
	**Reverse:** TCCACTGCCACAGTCAATCC		
BSP	**Forward:** AATGCAGAAGGCACCACAGA	241	NM_004967.3
	**Reverse:** AATTGTCCCCACGAGGTTCC		
OPN	**Forward:** GAAGTTCTGAGGAAAAGCAGC	161	NM_001040058.1
	**Reverse:** GGACTTACTTGGAAGGGTCTCT		
OCN	**Forward:** ATGAGAGCCCTCACACTCCT	180	NM_199173.4
	**Reverse:** TGGGGCTCCCAGCCATT		
RUNX2	**Forward:** CACTGGCGCTGCAACAAGA	157	NM_001024630.3
	**Reverse:** CATTCCGGAGCTCAGCAGAATAA		
Periostin	**Forward:** AAGCGCTTTAGCACCTTCCT	930	NM_006475.2
	**Reverse:** CTTCCTCACGGGTGTGTCTC		

#### Immunohistochemical Staining

Periodontal ligament stem cells (2.0 × 10^4^/well, second passage) were sub-cultured on 12-chamber slides (Costar; Corning Life Sciences, Tewksbury, MA, United States) and grown to 80% confluence. Cells were then fixed in 4% paraformaldehyde and blocked with phosphate buffered saline (PBS) containing 10% normal equine serum (Gibco; New Zealand) for 45 min at room temperature. Cells were then incubated with diluted primary antibody against scleraxis (1:500 dilution; D-14, Santa Cruz Biotechnology, United States) at 4°C overnight. Afterward, cells were washed with PBS, then incubated with fluorescein-conjugated goat anti-mouse polyclonal secondary antibody (Zenon Alexa Fluor 488, Life Technologies, United States) at room temperature in the dark for 45 min. DAPI (Invitrogen, United States) staining was then performed in the dark for 5 min. Slides were washed with PBS, and were analyzed using fluorescence microscopy (Nikon ECLIPSE TS-100; Tokyo, Japan). The scleraxis staining was done for all five PDLSC clones.

#### Flow Cytometry

In order to evaluate the mesenchymal phenotype in PDLSCs and the potential changes of the PDLSCs after being exposed to ECM membrane for 24 h, the surface antigens of PDLSCs were analyzed by flow cytometry. Cells were trypsinised and incubated in PBS for 60 min with fluorescein conjugated antibodies against CD45 (PerCP/Cy5.5, Biolegend), CD73 (Brilliant Violet421, Biolegend), CD90 (FITC, BD Pharmingen), and CD105 (PE, Biolegend). The flow cytometry analysis of cells was carried out using a Beckman Coulter MoFlo^®^ Astrios^TM^ flow cytometry system, with 500,000 events being counted for each case. Each procedure was repeated three times for each separate PDLSCs clone. PDLSCs co-cultured with ECM and PDLSCs alone underwent FACS separately to evaluate their immunophenotype and identify if ECM changed the expression of mesenchymal stem cell (MSC) phenotypic markers in PDLSCs.

### Determination of Cell Viability, Proliferation, and Cell Apoptosis

#### Viability

Periodontal ligament stem cells (5 × 10^3^ cells/well, second passage) were expanded *ex vivo* in 96-well plates and covered with ECM or type I collagen membranes, at 37°C for 24 h. PDLSCs viability was evaluated using the cell counting kit-8 (CCK-8) assay (Dojindo, Kumamoto, Japan); The assay was repeated for all five PDLSC clones separately as previously described (Wang et al., 2015).

#### Proliferation

Periodontal ligament stem cells (5 × 10^3^ cells/well, second passage) were expanded *ex vivo* in 96-well plates and covered with ECM or type I collagen membranes, at 37°C. At days 1, 3, 5, and 7 after cell seeding, cell viability was evaluated using the CCK-8 assay (Dojindo, Kumamoto, Japan), according to the manufacturer’s instructions. The assay was repeated five times for each sample to evaluate the number of viable cells.

#### Apoptosis

##### Hoechst 33342 staining

The DNA-specific fluorochrome Hoechst 33342 (Cell Signaling Technology) was used to analyze the nuclear morphology of cells following incubation on ECM and type I collagen membranes at 37°C. 24 h after incubation with the membranes, PDLSCs were seeded onto glass slides and fixed in 4% paraformaldehyde at room temperature for 30 min. Afterward, cells were stained with 10 μM Hoechst 33342 dye at room temperature for 20 min. Apoptotic nuclei were counted under a fluorescent microscope (Olympus BX-51, Tokyo, Japan). As a positive control for induction of apoptosis, 500 nM staurosporine (Sigma-Aldrich) was added to some samples and incubated for 5 h. Untreated cells were used as a negative control. Apoptotic cells (fragmented nuclei) were scored manually; at least 200 cells/time point were analyzed.

##### Flow cytometry

The rate of cellular apoptosis rate was observed by flow cytometry. PDLSCs were cultured with the ECM and type I collagen membranes at 37°C for 24 h. The rate of apoptosis was evaluated using a FITC Annexin V Apoptosis Detection Kit (BD Pharmingen), following standard protocols using the Beckman Coulter MoFlo^®^ Astrios^TM^ flow cytometry system. Cells negative for both PI and annexin V-FITC were considered viable. Cells positive for annexin V-FITC and negative for PI were considered apoptotic. Apoptosis progress was followed over four time points. Cells with a direct increase in Annexin V + /PI + staining without an intermediate step of Annexin V + /PI- were considered necrotic. The experiment was repeated three times for each separate PDLSCs clone.

### Determination of Cell Migration

Periodontal ligament stem cells (2.0 × 10^4^/well, second passage) were sub-cultured on six-chamber plates and grown to 90% confluence. A scratch experiment was carried out as the following steps: (1) scratch across the cells in each well diagonally; (2) rinse on half of the cells with PBS to wash them or to remove them from one of the halfs of each well; (3) placement of membranes to the washed area and were co-cultured with the cells for 24 h. The distance between the membranes and the cells edges were measured at five different sites at immediately after placement (0 h) and after 24 h co-culture by ImageJ software [National Institutes of Health (NIH), Bethesda, MD, United States].

### Determination of Cell Bio-Attachment

Periodontal ligament stem cells were covered with the surface of ECM or type I collagen membranes, and cultured for 24 h. Membranes were fixed with 4% paraformaldehyde for 30 min, and embedded using O.C.T compound (Tissue-Tek), serial 5 μm longitudinal sections of the ECM membrane and absorbable collagen membrane were cut and stained with hematoxylin-eosin (H&E) and DAPI (Invitrogen). Briefly, for H&E staining, the sections were placed on slides, dried at 37°C overnight, then rehydrated using a graded alcohol series, and then stained with hematoxylin for 3 min. Sections were rinsed in running tap water for 10 min until they turned blue and then stained with eosin for 3 min. For DAPI staining, sections were loaded DAPI solution for 5 min in the dark, washed with PBS and then were analyzed using fluorescence microscopy (Nikon ECLIPSE TS-100; Tokyo, Japan). The experiment was repeated for all five PDLSC clones separately.

### Cementoblastic/Osteogenic Differentiation of PDLSCs

Periodontal ligament stem cells (2.0 × 10^4^/well, second passage) were sub-cultured on six-chamber plates and grown for 5 days in α-minimum essential medium containing 15% fetal bovine serum, 50 μg/ml ascorbate, 100 U/mL penicillin and 100 mg/mL streptomycin. Mineralization medium was induced by supplementation of culture media with 10 mM β-glycerophosphate, media was replaced daily. ECM membrane and type I collagen membrane were added to the osteogenic medium separately. Cells cultured in mineralization medium but untreated with ECM membrane or type I collagen membrane were used as the control group. After induction for 7 days, alkaline phosphatase enzyme activity assay, Alizarin Red staining, and RT-PCR for gene expression analysis were performed.

### Alkaline Phosphatase Enzyme Activity Assay

TNAP enzyme activity was evaluated using the colorimetric substrate, nitro blue tetrazolium/5-bromo-4-chloro-3-indolyl phosphate (Sigma). Cells were fixed in 70% ethanol for 10–15 min at room temperature, air-dried and incubated with substrate for 1 h at 37°C. Cells were then rinsed with distilled water, air-dried, and visualized macroscopically for evidence of staining. For quantification, the plates were scanned, and the staining intensity was measured using the ImageJ software [National Institutes of Health (NIH), Bethesda, MD, United States].

### Alizarin Red Staining

Cells were rinsed with phosphate-buffered saline, fixed for 30 min in 100% ethanol, air-dried and then incubated with 0.5% Alizarin Red-S for 30 min. Cells were then washed with 70% ethanol and air-dried. The plates were scanned, quantification of the staining intensity was measured using the ImageJ software [National Institutes of Health (NIH), Bethesda, MD, United States].

### Statistical Analysis

Data are expressed as means ± SD. Between-group differences were compared using one-way ANOVA using SPSS software (version 13.0; SPSS, Chicago, IL, United States; statistical significance was at *P* < 0.05).

## Results

### Isolation and Characterization of PDLSCs

**FIGURE 1:**
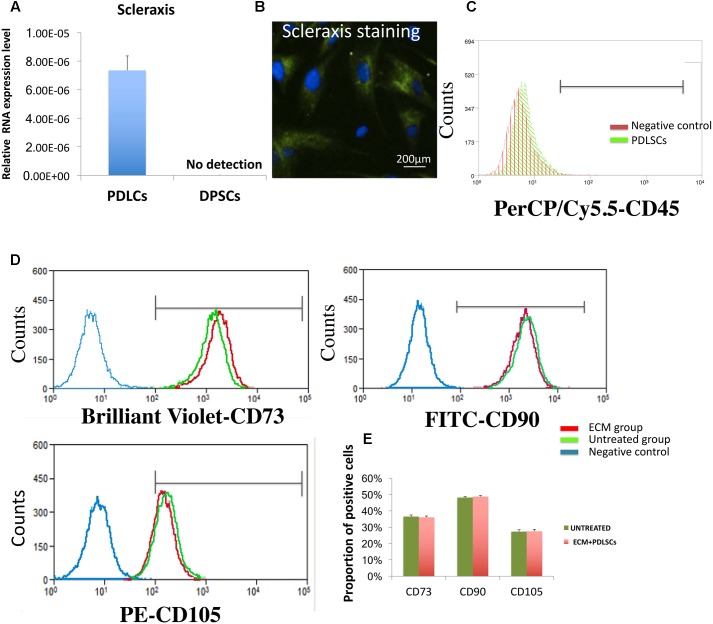
Biomarker characterization of PDLSCs. **(A,B)** RT-PCR and immune-histochemical staining in PDLSCs showing expression of scleraxis, a marker for PDL tissues. DPSCs have been used as a negative control. **(C)** Flow cytometry analysis revealed that PDLSCs lack expression the blood cell marker CD45. **(D)** Flow cytometry analysis revealed that PDLSCs express the MSC markers CD73, CD90, and CD105. No significant difference in the percentage of cells positive for MSCs markers between the ECM group and the untreated group. **(E)** Statistical analysis of the flow cytometry results.

RT-PCR and immuno-histochemical analysis confirmed that PDLSCs, but not DPSCs, were positive for scleraxis, a marker for tendon tissue which is also considered as a phenotypic marker of PDL cells (**Figures 1A,B**). Flow cytometric results showed a lack expression of CD45 which is a marker of hematopoietic cells (**Figure 1C**). In addition, PDLSCs expressed the MSC markers CD73, CD90, and CD105 after 24 h of culture with ECM membrane. No significant difference in the percentage of cells positive for MSCs markers between the ECM group and the untreated group was found and the flow cytometric analysis indicated that the characteristics of ECM-cultured cells remained unchanged in comparison to the control group (**Figures 1D,E**).

### Cell Viability, Proliferation, and Apoptosis

**FIGURE 2:**
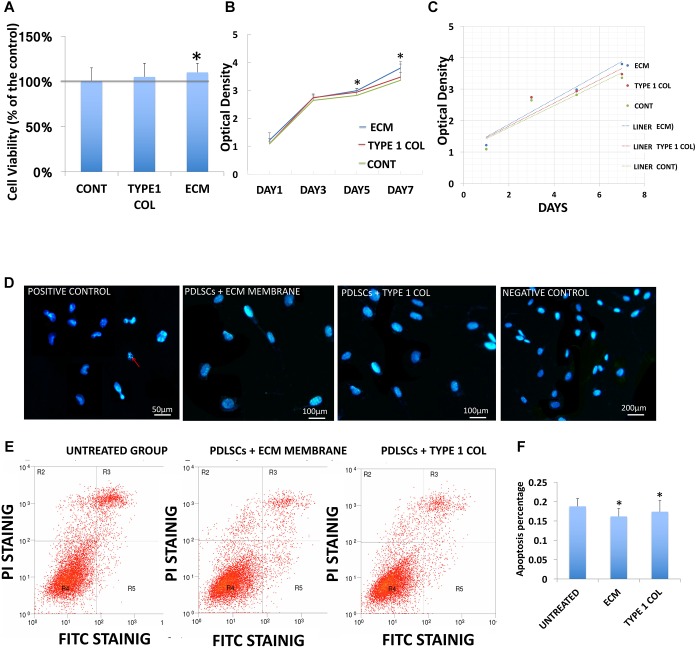
PDLSCs viability and apoptosis. **(A)** The viability of PDLSCs after 24 h of cell culture with ECM or type I collagen, is measured here as a percentage of live cells for the experimental groups and control group. This data shows statistically significant differences between ECM group and control group (^∗^*P* < 0.05). **(B)** The growth curve of PDLSCs after 1, 3, 5, and 7 days in culture with ECM, type I collagen membrane, or control show higher proliferation for ECM group at day 5 and 7. **(C)** The liner fit of the growth curve show a higher proliferation rate in ECM group. **(D)** Evaluation of cellular apoptosis by Hoechst staining 24 h. after incubation Hoechst staining was performed with ECM or type I collagen membrane to evaluate nuclear condensation (red arrow). No obvious apoptosis was observed in any of the three experiment groups. **(E)** FITC/PI staining detected by flow cytometric study showed the apoptosis rate derived from flow cytometric analysis. **(F)** The apoptosis rate was quantified by calculating the areas R2 + R3 + R5 in **D**, statistical analysis revealed that PDLSCs treated with ECM or type I collagen membrane have significantly lower apoptosis rates than the control group (^∗^ = statistically significant difference between control and experimental groups ECM and type I collagen membrane, *P* < 0.05).

The viability of PDLSCs cultured with ECM membrane or type I collagen membrane was evaluated. Both ECM and type I collagen membranes promoted cell growth but PDLSCs demonstrated significantly higher cell viability after incubation with ECM membrane compared to PDLSCs incubated with type I collagen membrane or the control group (**Figure 2A**).

Cell proliferation increased linearly from day one to day seven of culture in cells co-cultured with either type of membrane materials, and in the control group as well. However, the proliferation rate in cells co-cultured with ECM membrane was higher compared to the type I collagen membrane (**Figures 2B,C**).

The appearance of fragmented nuclei was used as an indicator of apoptosis. Hoechst 33342 staining showed no obvious apoptosis after cells were exposed to the culture media and ECM membrane or type I collagen membrane for 24 h (**Figure 2D**). The results of FITC/PI staining detected by flow cytometric study showed the overall apoptotic rate of PDLSCs cultured with ECM membrane or type I collagen membrane was significantly lower (*P* < 0.05) than the control group (**Figures 2E,F**).

### Cell Migration

**FIGURE 3:**
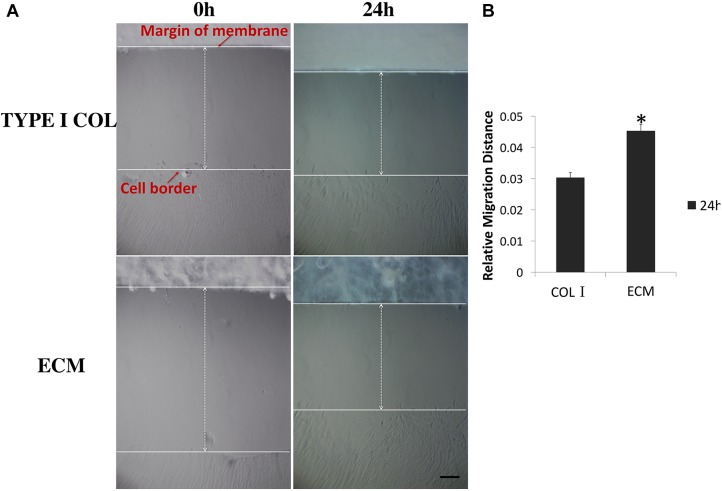
Measurement of PDLSCs cell migration. **(A)** Measurements of the relative migration distance at 0 and 24 h after wound scratching in the presence of ECM or type I collagen membrane were taken and representative photographs are shown here. **(B)** Differential relative migration distance, which is indicative of wound closure, is calculated between the two groups as mean ± SEM (*n* = 5) (^∗^ = statistically significant difference in migration distance between ECM and type I collagen membrane group, *P* < 0.05).

In a 24 h scratch assay, culture with ECM membrane promoted significantly greater cell migration of PDLSCs compared to incubation with type I collagen membrane (**Figures 3A,B**).

### Bio-Attachment of PDLSCs

**FIGURE 4:**
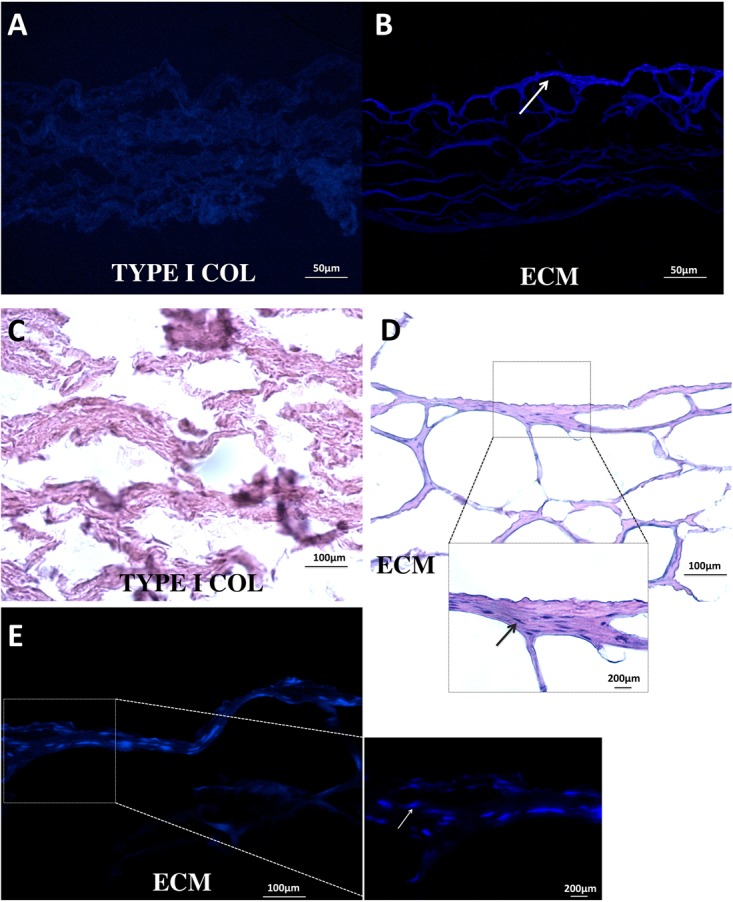
Bio-attachment of PDLSCs. After 24 h, ECM membrane showed a stronger bio-attachment capacity for PDLSCs than type I collagen membrane. PDLSCs were found distributed in the ECM membrane **(B–E)**; in contrast, no typical cell morphology was observed in the type I collagen membrane **(A,C)**.

Extracellular matrix membrane showed a significantly stronger capacity to induce PDLSC bio-attachment than type I absorbable collagen membrane (**Figures 4A–D**). PDLSCs were clearly observed in the ECM membrane (**Figure 4E**).

### Cementoblastic/Osteogenic Differentiation of PDLSCs

**FIGURE 5:**
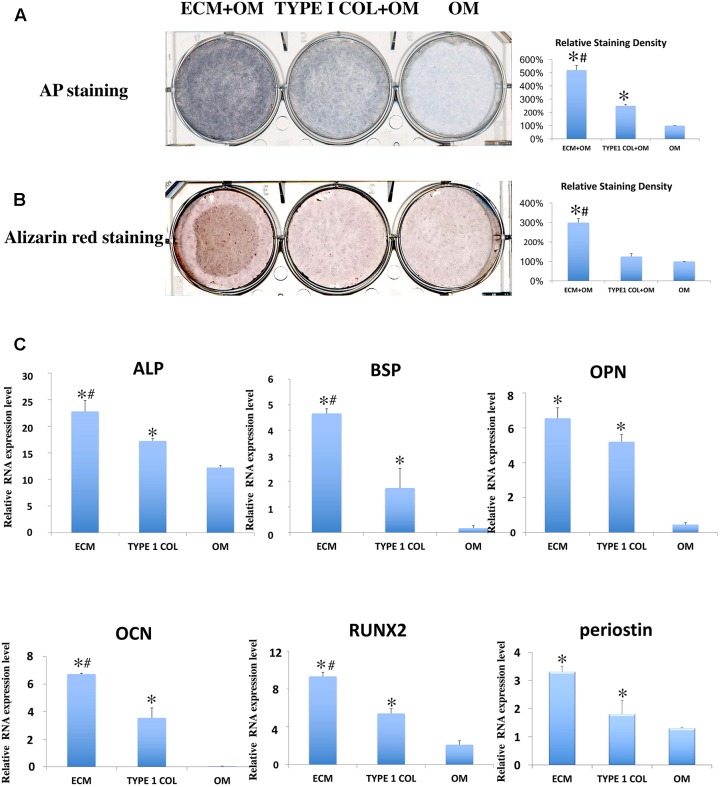
Cementoblastic/osteogenic differentiation of PDLSCs. Cementoblastic/osteogenic differentiation was evaluated by TNAP enzyme activity **(A)** and Alizarin Red **(B)** staining. The relative staining density was calculated between PDLSCs alone (OM: Osteoblastic medium) group and the two experimental groups (ECM + OM; TYPE 1 COL + OM) and was found statistically different being the highest in the ECM + OM group **(A,B)** (^∗^ = statistically significant difference found when compared to the control; ^#^ = statistically significant difference found between ECM and type I collagen membrane groups). **(C)** RT-PCR was also performed using primers for cementoblastic/osteogenic biomarkers ALP, BSP, OPN, OCN, and RUNX2 and relative RNA expression levels were analyzed by statistics (^∗^ = statistically significant difference were found between the PDLSCs alone group and the two experimental groups; ^#^ = statistically significant difference found between ECM and type I collagen membrane groups; *P* < 0.05).

After incubation for 7 days, ECM membrane with osteogenic differentiation medium, showed increased TNAP enzyme activity of PDLSCs when compared to incubation with type I collagen/osteogenic differentiation medium or to incubation with osteogenic differentiation medium alone (**Figure 5A**). ECM membrane also significantly promoted calcium deposition compared to type I collagen/osteogenic differentiation medium group or to the osteogenic differentiation medium alone (**Figure 5B**). The cementoblastic/osteogenic differentiation markers BSP, RUNX2, ALP, OPN, OCN, and periostin were also significantly Up-Regulated in the ECM (**Figure 5C**).

## Discussion

Periodontitis is a widespread dental infection disease affecting 47.2 percent of American adults (Eke et al., 2012) and causing severe periodontal defects in the root area of teeth. GTR using biodegradable membranes is a commonly used therapeutic approach that aims to enhance periodontal tissue regeneration. Although the exact mechanism of periodontal tissue regeneration under GTR remains unclear, is it hypothesized that the barrier provided by the Collagen I (COLI) membranes allows PDLSCs cell migration and regeneration of the infected periodontal tissues (Nyman et al., 1982; Gottlow et al., 1986). These COLI membranes are bio-inert supporting cell proliferation while minimizing cellular inflammation (Gentile et al., 2011) and are routinely used under well documented clinical protocols (Hämmerle et al., 1992; Alarrayed et al., 1995; Bornstein et al., 2007; Zubery et al., 2007; Friedmann et al., 2011). Studies suggest that COLI membranes also promote hemostasis, allowing early wound stabilization, nutrient passage, and fibroblasts recruitment through chemotaxis (Postlethwaite et al., 1978). However, the disadvantages of native collagen type I are primarily in the duration of barrier function, which cannot be strictly controlled, as loss of structural integrity and solubilization of the membrane via phagocytosis may precede the complete healing of the defect (Tal et al., 2008).

The research on dental tissue regeneration (Chatzistavrou et al., 2012) and on bactericidal and bioactive dental materials (Chatzistavrou et al., 2014; Kattan et al., 2015) is being largely explored in the recent years. In particular, the use of natural ECM as a biologic scaffold for dental tissue engineering applications has been suggested as a viable choice for a variety of clinical applications (Wang et al., 2015). In this study, we describe the effects of ECM membrane in PDLSCs cell behavior.

Periodontal ligament stem cells isolated from periodontal tissue were found to expresses scleraxis, a marker of PDL cells (Seo et al., 2004). *Ex vivo* expanded PDLSCs also expressed the MSC-related proteins CD73, CD90, CD105, and lacked expression of the CD45 which is considered a marker of hematopoietic cell lineage (Horwitz et al., 2005; Dominici et al., 2006). These characteristics indicated that the PDLSCs isolated and used in this study retained qualities of PDL mesenchymal stem cells (PDLSCs).

Therapeutic periodontal tissue regeneration relies on migration and differentiation of PDLSCs at the site of injury. Compared to type I collagen membrane, ECM membrane induced higher cell viability, more rapid proliferation rates, lower apoptosis percentage, and an improved capacity for bio-attachment of PDLSCs. These activities are important in facilitating the cementoblastic/osteogenic function of PDLSCs. Follow up studies will further evaluate ECM in clinical relevant scenarios using animal models of periodontal diseases.

The cementoblastic/osteogenic activity of PDLSCs was characterized by evaluating periodontal tissue differentiation biomarkers including TNAP enzyme activity, Alizarin red stain, and differential mRNA expression. Both TNAP and Alizarin red are commonly used stains to measure cementoblastic/osteogenic activity in bone and dentin-forming tissues (Hong et al., 2010; Nam et al., 2011) and ECM strongly enhanced PDLSCs TNAP activity and Alizarin staining. Furthermore, RT-PCR analysis showed that PDLSCs incubated with ECM membrane showed significant Up-Regulated mRNA expression of all these markers when compared to incubation with type I collagen membrane or to untreated cells. These data confirm that ECM membrane has superior biological properties compared to COLI membrane that significantly promote PDLSCs cell proliferation, differentiation, and mineral formation. We propose that these enhanced properties are the result of released growth factors from ECM into the periodontal tissues micro-environment as it has been shown previously in other systems (Voytik-Harbin et al., 1997; Hodde et al., 2001), the generation of bioactive cryptic peptides (Agrawal et al., 2011), or signaling molecules contained within recently identified matrix bound nano-vesicles (Huleihel et al., 2016).

The molecular composition of ECM includes several structural proteins such as collagen type I, glycosaminoglycans, proteoglycans, glycoproteins as well as numerous cytokines and growth factors which are shown to guide cell behavior (Timpl, 1996; Li et al., 2004). Among molecular factors, and biologic peptides found in ECM several have been investigated for bone healing and epithelial tissue regeneration. Furthermore, ECM displays a large degree of conservation among species (Constantinou and Jimenez, 1991; Exposito et al., 1992). These similarities in ECM molecular composition and the conservation among species highlight the fundamental importance of the ECM in cell homeostasis and tissue repair mechanisms (Badylak, 2005).

Type I collagen is widely used as a biologic scaffold for therapeutic applications, including in GTR therapy. In this study, we showed that ECM has a better bio-activity compared to type I collagen membrane alone due potentially to the complex components present in ECM in addition to Collagen I as discussed above. Therefore, the advantage of tissue-extracted ECM for tissue regeneration may come from the presence of molecular factors in amounts and forms similar to the ones that exist in nature. We therefore hypothesize that these distinct characteristics of ECM make it a more suitable material for use in periodontal tissue regeneration than traditional type I collagen membrane.

In conclusion, our study demonstrates that ECM membrane enhances PDLSCs proliferation and cell viability, and cell differentiation suggesting that ECM scaffolds has enhanced periodontal tissues regenerative properties when compared to COLI membranes. Additional *in vivo* studies are required to further confirm this hypothesis and provide a strong foundation for follow up clinical trials. Our study expands the field of regenerative dentistry and provides the foundation for future studies on the clinical application of ECM material membranes.

## Author Contributions

YW, PP, SP, and LG provided the design of the study. YW carried out the experiment and analyzed the data. SB and DF provided expertise with ECM. YW and YZ drafted the article and revised it critically for important intellectual content. MQ and PP made the final approval of the version to be submitted. MQ and PP were equally contributed senior authors.

## Conflict of Interest Statement

The authors declare that the research was conducted in the absence of any commercial or financial relationships that could be construed as a potential conflict of interest.
